# Importance and usage of chromosomal microarray analysis in diagnosing intellectual disability, global developmental delay, and autism; and discovering new loci for these disorders

**DOI:** 10.1186/s13039-018-0402-4

**Published:** 2018-09-24

**Authors:** Ahmet Cevdet Ceylan, Senol Citli, Haktan Bagis Erdem, Ibrahim Sahin, Elif Acar Arslan, Murat Erdogan

**Affiliations:** 1 Trabzon Kanuni Training and Research Hospital, Medical Genetics Unit, Trabzon, Turkey; 2 Ankara Yıldırım Beyazıt University, Ankara Atatürk Training and Research Hospital, Department of Medical Genetics, Ankara, Turkey; 3 Ankara Diskapi Yildirim Beyazit Training and Research Hospital, Medical Genetics Unit, Ankara, Turkey; 4 Karadeniz Technical University, School of Medicine, Department of Child Neurology, Trabzon, Turkey; 5 Kayseri Training and Research Hospital, Department of Medical Genetics, Kayseri, Turkey

**Keywords:** Chromosomal microarray, Intellectual disability, Global developmental delay, Copy number variations, 22q11.2 homozygote duplication

## Abstract

**Background:**

Chromosomal microarray analysis is a first-stage test that is used for the diagnosis of intellectual disability and global developmental delay. Chromosomal microarray analysis can detect well-known microdeletion syndromes. It also contributes to the identification of genes that are responsible for the phenotypes in the new copy number variations.

**Results:**

Chromosomal microarray analysis was conducted on 124 patients with intellectual disability and global developmental delay. Multiplex ligation-dependent probe amplification was used for the confirmation of chromosome 22q11.2 deletion/duplication. 26 pathogenic and likely pathogenic copy number variations were detected in 23 patients (18.55%) in a group of 124 Turkish patients with intellectual disability and global developmental delay. Chromosomal microarray analysis revealed pathogenic de novo Copy number variations, such as a novel 2.9-Mb de novo deletion at 18q22 region with intellectual disability and autism spectrum disorder, and a 22q11.2 region homozygote duplication with new clinical features.

**Conclusion:**

Our data expand the spectrum of 22q11.2 region mutations, reveal new loci responsible from autism spectrum disorder and provide new insights into the genotype–phenotype correlations of intellectual disability and global developmental delay.

## Background

Advancements in molecular technology, such as chromosomal microarray analysis (CMA), has led to the discovery of new microdeletions and microduplications in patients with intellectual disability (ID), global developmental delay (GDD), epilepsy, and congenital anomalies (CA). ID and GDD are clinically heterogeneous neurodevelopmental disorders seen in 1–3% of children [1]. CMA is a first-tier test in the evaluation of individuals with ID and GDD with the diagnostic yield ranging from 5 to 20% varying based on population examined [2, 3]. The International Standard for the Consortium of Cytogenomic Array recommended CMA as a first-stage cytogenetic diagnostic test for patients with CA and ID / GDD.

CMA can detect well-known microdeletion syndromes. It also contributes to the identification of genes that are responsible for the phenotypes in the new copy number variations (CNVs). Chromosome 22q11.2 deletion syndrome and 22q11.2 duplication syndrome are good examples for microdeletion/microduplication syndromes. Chromosome 22q11.2 duplication syndrome was first explained in 1999, but research continues to explore new phenotypes of this syndrome [4]. When there are three copies of the 22q11.2 region, it is called 22q11.2 duplication, whereas four copies of this region are referred to as the tetrasomy of 22q11.2 region. When there are two copies of 22q11.1 region in both alleles, this is referred to as 22q11.2 homozygote duplication. If there are three copies in one allele and one copy in the other, it is called 22q11.2 triplication.

Autism is a childhood disorder that expresses itself through core problems in communication and social interaction skills, along with the presence of stereotypical behaviors. Autism spectrum disorder (ASD) does not have a fixed pattern since a number of different genes have been reported as responsible for this disorder [5]. CMA studies have a high potential in revealing novel gene-phenotype associations in relation to this disorder. In this study, new loci for autism have been discovered which will help us better understand the cause of the disease.

Herein, we present the diagnostic rates for CNVs, and the aberrations, with major clinical findings detected by CMA in a group of 124 Turkish patients with ID and GDD. We also present a case with 22q11.2 homozygote duplication and new phenotypic findings as a result. Our study will help better explain the genotype-phenotype associations of the 22q11.2 region.

## Methods

### DNA extraction

Genomic DNA of family members was extracted according to the manufacturer’s standard procedure using the MagNA Pure Compact Nucleic Acid Isolation Kit I (Roche Diagnostic GmbH, Mannheim, Germany).

### Microarray analysis

Affymetrix CytoScan Optima® chips were used to perform CMA in 124 patients with ID and GDD at Trabzon Kanuni Training and Research Hospital. Data analysis was performed using Chromosome Analysis Suite 3.1 software. Data were presented as minimum coordinates (sequence positions of the first and last probes within the CNV) in the NCBI37/hg19 genome assembly. Variants were evaluated based on phenotype and using standard in silico tools [6]. The analysis and interpretation of the obtained results were performed by using public genomic databases, such as UCSC, OMIM, DGV, DECIPHER, CLINGEN.

### MLPA (multiplex ligation-dependent probe Amplication) analysis

MLPA was performed as suggested by the manufacturer (MRC-Holland®, Amsterdam, The Netherlands). The SALSA® MLPA® probemix P250 DiGeorge was used for the confirmation of chromosome 22q11.2 deletion/duplication. The standard deviation of all probes in the reference samples were < 0.10 and the dosage quotient (DQ) of the reference probes in the patients’ samples were between 0.80 and 1.20. DQ of the probes were between 0.40 and 0.65 for heterozygous deletion. DQ of the probes were between 1.30 and 1.65 for heterozygous duplication. DQ of the probes were between 1.75 and 2.15 for heterozygous triplication or homozygous duplication.

## Results

Between May 2016 and April 2017, 124 patients were examined at the department of genetics for GDD/ID and CA. There were 73 males and 51 females. The age of the patients ranged from 15 days to 17 years. The Denver Developmental Test was used with patients up to 3 years of age, whereas the Standfort Benet Test was used for patients 4–6 years of age, and the WISC-R test for patients older than 6. This study was conducted with patients who were not diagnosed with any syndromes previously and CMA was performed as the first-tier test on the subjects. Table 1 shows the clinical features of these cases and the CMA results.

**Table 1:** Clinical findings of 23 patients and CNV status

Case	Age	Region	OMIM Phenotype (number)	Comment	Type/Size	Inheritance	Genes involved	Likely pathogenic genes	Intellectual Disability	Developmental Delay	Dysmorphic features	Other findings
1	1,5 y	chr1:849466–3,152,968	1p36 deletion syndrome (607872)	Path	Del 2.3 Mb	De novo	76	DVL1, ATAD3A, GNB1, GABRD, SKI, PEX10, PRDM16	N/A	+	prominent forehead, pointed chin, deep-set eyes, straight eyebrows	dilated cardiomyopathy, epilepsy
2	4 m	chr1:145927661–148,588,367	1q21.1 deletion syndrome (612474)	Path	Del 2.6 Mb	Mot her	31	NBPF10, HYDIN2, NBPF12, PRKAB2, FMO5, CHD1L, BCL9, ACP6, GJA5, GJA8, GPR89B, NBPF11, PPIAL4A, NBPF14, NBPF9	N/A	N/A	wide nasal bridge, bulbous nose and retrognathia.	pectoral muscle hypoplasis, radius and humerus hypoplasis, short and curved ribs
3	6 y	chr1:145607915–146,497,779		UCS/L-Path	Del 0.9 Mb	De novo	14	GPR89A, PDZK1, CD160, POLR3C, NBPF12	+	+	strabismus, hypertelorism	congenital hypothyroidism
4	2,5y	chr2:10266562–16,826,500		Path	Dup 6.5 Mb	De novo	20	ODC1, LPIN1, MYCN, NBAS,	N/A	+	prominent forehead, retrognathia, broad eyelashes, cupped ear, uplifted lobe	dysplastic aortic valve, hydrocephalus, cerebral atrophy
5	8 m	chr2:222686398–226,097,873		Path	Del 3.4 Mb	De novo	16	PAX3, AP1S3, MRPL44, CUL3	N/A	+	prominent forehead, hypoplastic alae nasi, epicanthal fold	
6	14,5 y	chr6:6806969–13,794,521		Path	Del 6.9 Mb	De novo	46	DSP, TFAP2A, GCNT2, MAK, GCM2, EDN1, TBC1D7	+	+	dolicocephaly, facial asymmetry, open mouth, depressed nasal tip, absent ear lobe,	atrial septal defect, bilateral cleft lip
7	8,5 y	chr6:70373236–79,654,154, chr8:78808754–81,835,864		Path Path	Del 9.2 Mb/Del 3 Mb	De novo	49	LMBRD1, COL9A1, RIMS1, KCNQ5, KHD3CL, MYO6,MTO1, SLC17A5, COL12A1,IMOG1	+	+	long face, wide mouth, high arched palate	potent ductus arteriosus, renal agenesis, pes equinovarus
8	4 m	chr6:151310706–170,919,482		Path	Del 19.6 Mb	De novo	115	SYNE1,ESR1, ARİD1B, LPA, PDE10A,T, ERMARD,TBP,	N/A	+	sunset eye sign, low set ears, pointed chin	hydrocephalus, cerebral atrophy
9	1 m	chr6:163378290–16,709,184, chr6:160713926–16,287,570, chr6:141494387–144,877,906		Path UCS/L-Path Path	Dup 3.7 Mb Del 2.1 Mb Del 3.3 Mb	De novo	17 8 19	PACRG, QKI, PDE10A, T, MPC1, RPS6KA2, SLC22A3, LPAL2, LPA, PLG, MAP3K4, AGPAT4, PARK2, NMBR, VTA1, GPR126, HIVEP2, AIG1, PEX3, PLAGL1, HYMAI, STX11, UTRN	N/A	+	upslanting palpebral fissures, round face	neonatal diabetus, hypotonia, deafness
10	9 y	chr14:103255460–107,285,437		Path	Del 4 Mb	De novo	56	TRAF3, APOPT1, RCC3, INF2, AKT1, BRF1,IGHM	+	+	long face, pointed chin, anterverted ears, epicanthal fold	–
11	11,5 y	chr14:99528241–107,285,437		Path	Dup 7.7 Mb	De novo	183	YY1, DYNC1H1, TECPR2, APOPT1, XRCC, ADSSL1, AKT1, ZBTB42, BRF1, IGHG2,	+	+	facial asymmetry, downslanting palpebral fissures, prognathism, macrocephaly	strabismus, ptosis,
12	5 y	chr15:22770421–30,295,864		Path	Dup 7.5 Mb	De novo	131	MKRN3, MAGEL2, NDN, SNRPN,UBE3A,GABRB3,OCA2, HER2	+	+	hypertelorism, depressed nasal bridge,	unilateral deafness, autism spectrum disorder
13	6 y	chr15:22770421–23,276,605		UCS/L-Path	Del 0.5 Mb	De novo	7	NIPA1, NIPA2, CYFIP1, TUBGCP5	+	+	hypertelorism, short palpebral fissures, blepharophimosis	corpus callosum agenesis, hypothyroidism
14	15 y	chr16:89342189–89,552,394	KBG Syndrome (148050)	Path	Del 0.2 Mb	De novo	2	ANKRD11	+	+	long and triangular face structure, large, protruding ears	degenerative myopia
15	13,5 y	chr18:136226–6,992,327		Path	Del 6.8 Mb	De novo	41	SMCHD1, LPIN2, TGIF1, LAMA1	+	+	hypertelorism, broad nasal bridge	obesity
16	10 y	chr18:59720983–78,014,123		Path	Del 18.2 Mb	De novo	72	PIGN,TNFRSF11A, BCL2, KDSR, SERPINB7, RTTN, CYB5A, TSHZ1, CTDP1, XNL4A	+	+	frontal bossing, deep set eyes, depressed nasal bridge	sensorineural deafness, strabismus
17	3 y	chr18:67847004–70,771,041		UCS/L-Path	Del 2.9 Mb	De novo	6	RTTN, SOCS6, CBLN2, NETO1	N/A	–	hypertelorism, broad nasal bridge	autism spectrum disorder
18	13 y	chr18:65852206–76,107,497		Path	Del 10 Mb	De novo	32	RTTN, SOCS6, CBLN2, NETO1, CYB5A, TSHXZ1, GALR1	+	+	frontal bossing, deep set eyes, protruding ears	feeding difficulties
19	9 m	chr19:11284538–13,555,660		UCS/L-Path	Del 2.2 Mb	De novo	78	KANK2, DOCK6, EPOR, PRKCSH, ACP5, MAN2B1, RNASEH2A, KLF1, CALR, NFIX, LYL1, NACC1, CACNA1A	N/A	+	deep set eyes, micrognatia	hypotonia
20	5 y	chr22:18917030–21,465,662	DiGeorge syndrome (188400)	Path	Del 2.5 Mb	De novo	66	PRODH, SLC25A, CDC45, GPİBB, TBX1, COMT, TANGO2, RTN4R, CARF2, PI4KA, SERPIND1,	+	+	hypertelorism, blunted nose, high arched palate,	tetralogy of fallot
21	5 y	chr22:19004771–21,443,283	22q11.2 microdup lication syndrome (608363)	Path	Dup 2.4 Mb	Father	63	SLC25A, CDC45, GPİBB, TBX1, COMT, TANGO2, RTN4R, CARF2, PI4KA, SERPIND1,	+	+	frontal bossing, synophrys, pytosis,	strabismus,
22	11,5 y	chr22:19077926–21,804,886	22q11.2 microdup lication syndrome (608363)	Path	Dup 2.7 Mb	De novo	65	SLC25A, CDC45, GPİBB, TBX1, COMT, TANGO2, RTN4R, CARF2, PI4KA, SERPIND1,	–	–	no dysmorphic features	loss of cognitive functioning
23	11 y	chr22:18917030–21,421,425	22q11.2 microdup lication syndrome (608363)	Path	Hom dup 2.5 Mb	Mother and father	66	PRODH, SLC25A, CDC45, GPİBB, TBX1, COMT, TANGO2, RTN4R, CARF2, PI4KA, SERPIND1,	+	+	Round face, broad nasal bridge, hypertelorism, downslanting palpebral fissures, long philtrum, overfolded helix	patent ductus arteriosus, hypoplasia of clivus

**Fig. 1:**
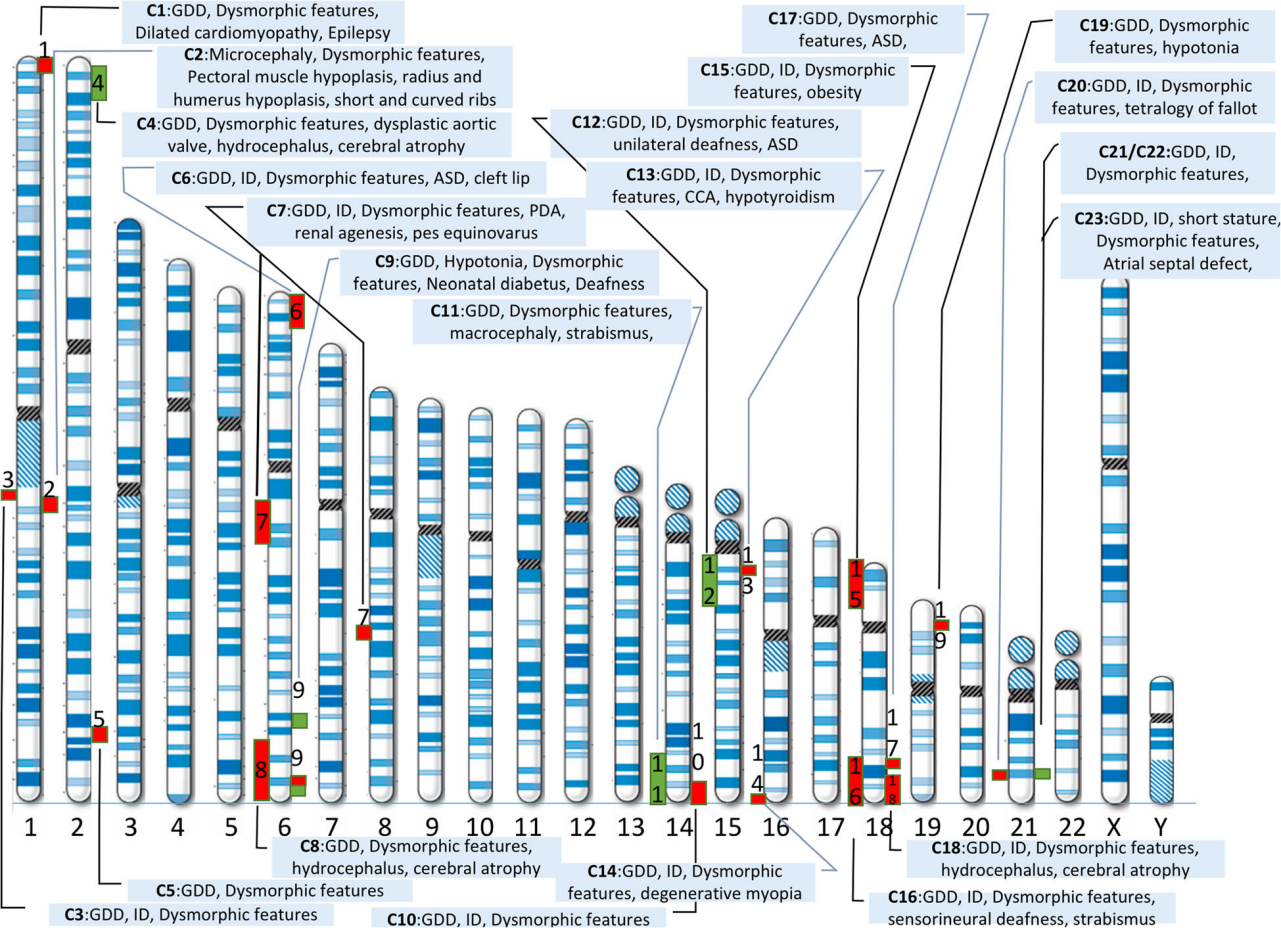
Presentation of CNV’s on Chromosome Ideogram with Major Clinical Findings. (Deletions: red, duplications: green) The figure was created via Microsoft® PowerPoint software

In 23 individuals (from 23 families), CMA analysis revealed CNVs including 6 microduplications (2p25p24, 22q11.2 [2 cases], 2p25, 14q32, and 15q11.2), 14 microdeletions (1p36.3, 1q21 [2 cases], 2q36, 6p25.1p23, 6q25q27, 14q32, 15q11.2, 16q24, 18p11.3, 18q22, 18q21q23, 19p23, and 22q11.2) and 1 homozygote duplication identified in same locus (22q11.2) in a patient (Fig. 1).

Twenty one of the 26 CNVs were interpreted as pathogenic, whereas 5 of the 26 CNVs were interpreted as having uncertain clinical significance (UCS); likely pathogenic in accordance with the clinical findings about the patients and literature [7]. 7 of 26 CNVs were associated with well-known microdeletion/ microduplication syndromes. Interestingly, multiple CNVs have been identified in two of the patients including 2 different deletions (6q13q14 and 8q21.3) in one and 1 deletion (6q25) and 2 duplications (6q24.1 and 6q26) in another (Table 1).

The length of 17 of the CNVs were below 5 Mb and they could not be detected with conventional karyotyping. The length of 7 of the CNVs were between 5 and 10-Mb. It is worth to note that siblings, whose parents were in consanguineous marriages and carried 22q11.2 duplication, were also diagnosed with 22q11.2 homozygote duplication. 23 of 26 CNVs were de novo. Eight out of 23 families had consanguineous marriages. Only two of the case studies are presented below in detail, however, the summary of all clinical features and mutations observed in patients can be found in Table 1.

### Region 22q11.2 duplication/ homozygote duplication family (cases 23 in Table 1)

**Fig. 2:**
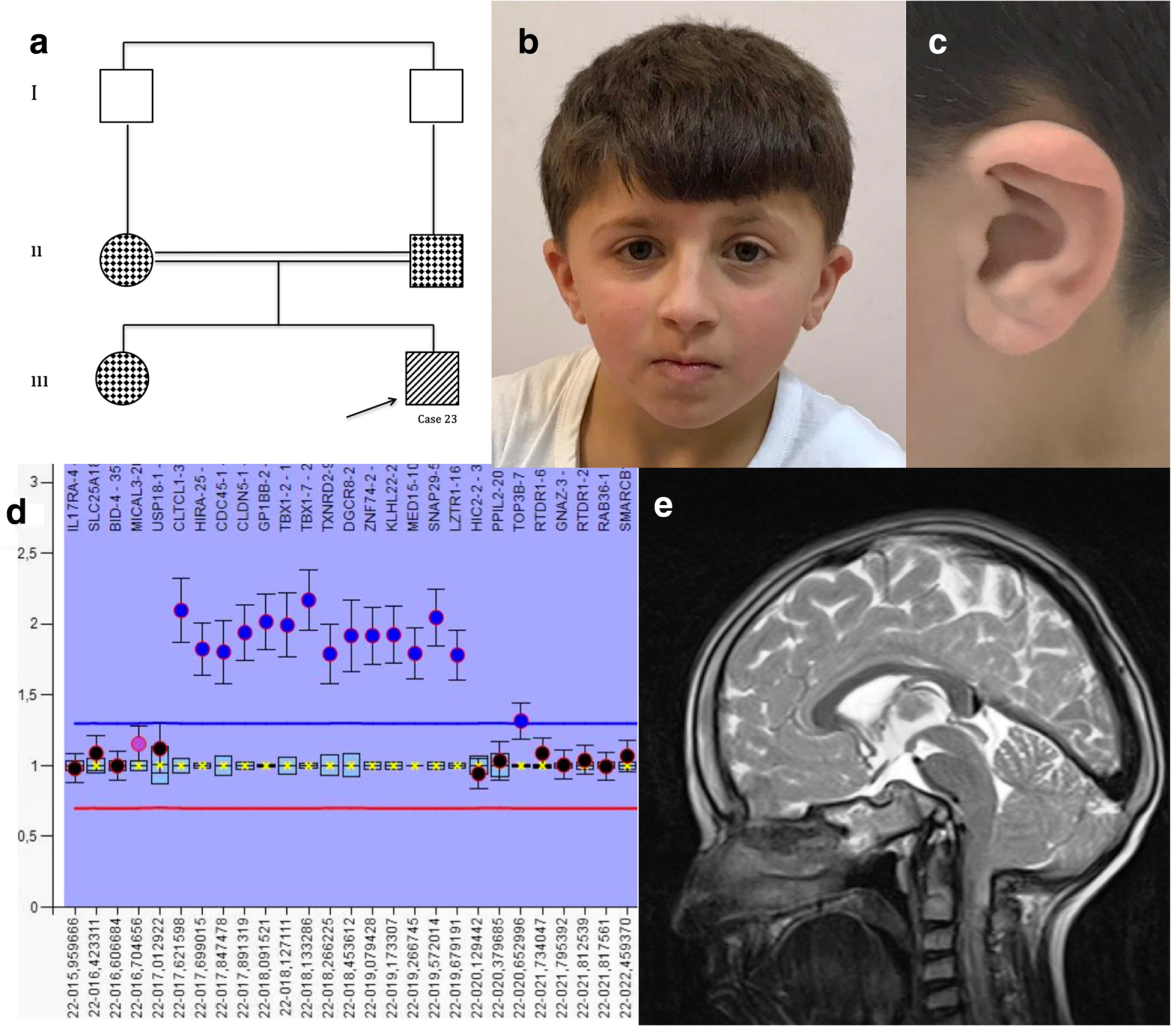
Case 23 with 22q11.2 homozygote duplication. **a** Pedigree of the family. **b** Dysmorphic features of the case 23. **c** Note the overfolded helix. **d** MLPA shows tetrasomy of 22q11.2 region. **e** T2 weighed MRI shows basillar impression and hypoplasia of clivus

An 11-year-old male patient (case 23) was diagnosed with learning disabilities. He started to walk in his first year and began speaking two-word sentences at the age of two. He was unable to learn how to read and write in his native language in the first year of elementary school, and was not able to keep up with his classmates. His history revealed that he had been hospitalized during the neonatal period due to high indirect bilirubinemia. His parents have a consanguineous marriage (Fig. 2a).

At his neurological examination, head circumference was 52 cm (25th–50th percentile), height was 121 cm (<3rd percentile), and weight was 25 kg (<3rd percentile). Round face, a broad nasal bridge, hypertelorism, downslanting palpebral fissures, long philtrum, and over-folded helix were observed (Fig. 2b). Hyperactivity and attention deficit disorder were not detected. No pyramidal system involvement was present, and reflexes were normoactive. Cerebellar system examinations were normal except for dysmetria and dysdiadochokinesia. Tandem gait exhibited no abnormality. Echocardiography revealed small patent ductus arteriosus. T2 weighed MRI shows basillar impression and hypoplasia of clivus. Odontoid process was measured approximately 15 mm above the Chamberlain line (Fig. 2e). Serum electrolytes, electroencephalography, and abdominal ultrasonography were normal. Psychometric evaluation revealed borderline mental disability. 2.5 Mb triplication or homozygote duplication was detected at 22q11.2/ Di George Syndrome region with CMA. Both his mother and father had the duplication of same region. The results were in accordance with the MLPA analysis (Fig. 2d).

The patient’s sister is 17 years old. She started to walk in her first year and began speaking two-word sentences at the age of 18 months. She was able to learn how to read and write in her native language in the first year of elementary school but she failed in mathematics. It was reported that she spoke less than her classmates and struggled building friendships. At her neurological examination, the circumference of her head was 54 cm (25th–50th percentile), her height was 155 cm (25th–50th percentile), and weight was 45 kg (10th–25th percentile). Hypertelorism, broad nasal bridge, downslanting palpebral fissures were observed. No pyramidal system involvement was present, and reflexes were normoactive. Echocardiography and brain MRI was normal. Psychometric evaluation revealed borderline mental disability. 2.5 Mb duplication was detected at 22q11.2/ Di George Syndrome region with CMA. The results were validated with MLPA analysis.

The patient’s father is 48 years old. He is a primary school graduate and works in the transportation sector. There were no neuropsychiatric problems observed other than sudden irritation. The mother is 44 years old. She is a primary school graduate. She is a housewife and is interested working in her own garden. She is capable of doing daily work, but has difficulty doing arithmetic calculations. Both the mother and the father had the duplication of the same 22q11.2/ Di George Syndrome region with CMA. The results were validated with MLPA analysis.

### Case 17 with a new locus for ASD

A 3-year-old male patient (case 17) was diagnosed with speech delay. He started to walk in his first year and began speaking by using three words at two and half years of age. At neurological examination, head circumference was 48 cm (10th percentile), height was 93 cm (10th–25th percentile), and weight was 13 kg (10th–25th percentile). Hypertelorism, broad nasal bridge, micrognatia were observed. Hyperactivity and poor eye contact were detected. No pyramidal system involvement was present, and reflexes were normoactive. Cerebellar system examinations were normal. Serum electrolytes, electroencephalography, and abdominal ultrasonography findings were within normal limits. He had stereotypical behaviors, deficits in communication, and autism spectrum disorder, which was diagnosed at 3 years of age. Denver developmental screening test showed one-year delay in speech and social skills. CMA revealed 2.9-Mb de novo deletion at 18q22 region. *RTTN, SOCS6, CBLN2, NETO1* genes were located at the deleted region.

## Discussion

Global developmental delay is the term that is used to describe children (aged 5 years or younger) who have demonstrated several significant delays in the following areas: cognitive, speech, social/personal, fine/gross motor, and daily activities. Intellectual disability is a disorder with intellectual and adaptive deficits and can be diagnosed after the age of five [5]. CMA is a first-tier test in the evaluation of individuals with ID and GDD which provides opportunities to discover new ID/GDD associated syndromes, and helps uncover the genetic background of many syndromes by revealing genetic heterogeneity and by identifying new loci for novel candidate genes [3]. The widespread use of CMA allows patients to be diagnosed and provides families with guidance in genetic counseling.

This is the first study shows the diagnostic rate of chromosomal abnormalities in the northern part of Turkey, with yield of 18.55%, which is consistent with the results of previous studies (5–35%) [2, 3]. It is important to point out that our diagnostic rate is higher than the rate of other studies performed on a similar platform in Turkey (12–13.6%) [8, 9]. We found 26 CNV’s in 23 patients, which indicates the importance of the CMA. Different diagnostic rates for different publications are related to the choice of patient group. If more patients were involved in the study, the rates could differ.

We also included parents in our study, and 23 of 26 CNVs were de novo. Although 8 out of 23 families had consanguineous marriages, our analyses revealed de novo variations in these families rather than homozygous mutations. In Turkish society, where the consanguineous marriage is common, it is necessary to investigate chromosomal abnormalities due to this phenomenon. We studied such a family, where mother and father were related and had a chromosome 22q11.2 duplication. The family had a girl with learning disability and was diagnosed with 22q11.2 duplication, and a boy (case 23) with milder intellectual disability, dysmorphic features and short stature, who was diagnosed with tetrasomy of 22q11.2 region. In case 23, parents’ diagnoses support the duplication of region 22q11.2 in both alleles, which can be described as homozygous duplication. Case 23 is the fifth patient diagnosed with 22q11.2 tetrasomy in the literature [4, 10, 11] and the third patient diagnosed with 22q11.2 homozygote duplication (Table 2). In other published 22q11.2 tetrasomy cases, three copies of an allele were reported. Bi et al. reported two cases with 22q11.2 homozygote duplication. However, there are other genomic changes, outside the 22q11.2 region, which could affect the phenotype of these patients [11]. As those two patients have region 22q11.2 homozygote duplication, case 23 can be added to this list as the third patient with a similar situation. Although our patient has cognitive deficiency and dysmorphic facial features in common with the other three cases, it is important to note that our patient does not suffer from hearing loss unlike the other three cases (Table 2). However, while other triplications may be three copies of a parental tract, it should be noted that our patient’s mother and father have duplication of the region. Bi et al. reported that there was no phenotypic difference between duplication of 22q11.2 region and triplication of 22q11.2 [11]. While the T2 weighed MRI of the case 23 shows basillar impression and hypoplasia of clivus, hypoplasia of clivus has not been reported in the other region 22q11.2 triplication or duplication cases.

**Table 2:** Clinical features of patients with tetrasomy of region 22q11.2

Features	Yobb et al. 2005	Bi et al. 2012	Vaz et al. 2015	This study (case 23)
22q11.2 region	4 copies (3/1)	4 copies (2/2)	4 copies (3/1)	4 copies (2/2)
Age at last evaluation	8 years	29 mounts	20 years	11 years
Gender	Female	Male	Female	Male
Heart defect	–	–	+	+
Velopharyngeal insufficiency	–	–	+	–
Hearing impairment	+	+	+	–
Failure to thrive	–	–	–	+
Sleep apnea	–	N/D	+	–
Urogenital abnormalities	–	N/D	+	–
Cognitive deficits	+	+	+	+
Psychiatric disorders	–	N/D	+	–
Behavioural problems	+	N/A	–	–
Headache	–	N/D	+	–
Recurrent infections	+	–	–	–
Hand/foot abnormality	+	+	–	–
Dysmorphic and other features	Epicanthal folds, Broad nasal bridge, Left ear pit, Secondary hearing impairment	Left preauricular pit, Plagiocephaly, Facial asymmetry, Teeth abnormality, Hypoplastic iris and corectopia	Long narrow face, Epicanthal folds, Hypertelorism, Downslanting palpebral fissures, Prominent nose, Long philtrum, Dental cavities, Retrognathia,	Round face, Hypertelorism, Downslanting palpebral fissures Broad nasal bridge, Long philtrum, Overfolded helix, Hypoplasia of clivus

Case 17, who was diagnosed with autism spectrum disorder, had a 2.9 Mb deletion at 18q22 region. *RTTN, SOCS6, CBLN2, NETO1* genes are located at this region. *RTTN* homozygote mutations are associated with microcephaly, short stature, and polymicrogyria with seizures (OMIM 614833). [12]. Homozygote mutations of this gene also affect brain migration and volume [12]. Our patient had a deletion of 1-10th exons with milder phenotype. The MRI was normal and he had speech delay and autism spectrum disorder. These results show that the *RTTN* gene heterozygous mutations could be responsible for ASD.

*SOCS6* gene, which encodes Suppressor of Cytokine Signaling 6 protein, was deleted in case 17. The SOCS6 gene has not yet been associated with a disease, however it may be related to syndromic obesity [13]. Although this gene is deleted in our patient, he is not yet obese. Even though *SOCS6* and *SOCS7* have been reported to be necessary for cortical neuron migration [14], there was no evidence of migration defect in the MRI of our patient. The other gene in the deletion region in case 17, which may be important for ASD/ID, is *CBLN2. CBLN2* gene encodes Precerebellin 2. Common variants of *CBLN2* are associated with increased risk of pulmonary arterial hypertension [15]. It is noteworthy that *CBLN2* expression is highest in cerebral cortex and hypothalamus of mouse reference [16].

Neuropilin and Tolloid-Like 1 (*NETO1*) gene was deleted in case 17. *NETO1* is a CUB domain-containing transmembrane protein, which have been reported to immuno-precipitate with assembled NMDA receptors via GluN2A or GluN2B subunits [17]. Ng D et al. have shown that *NETO1* plays a critical role in maintaining the delivery or stability of NR2A-containing NMDARs at CA1 synapses [18].

Cody et al. reported that genes located distal to 18q were associated with ASD, and *NETO1* was among these genes [19]. The only common point between the deletion in our patient and the region that Cody et al. have reported is the *NETO1*. O’Donnell et al. also reported that *NETO1* and *FBXO15* genes in region 18q22.3 may be risk factors for ASD [20]. When the synaptic plasticity is thought to be important in the development of ASD, heterozygous *NETO1* deletions may be considered as a risk factor for ASD. After doing a thorough evaluation of the literature, particularly the studies with mice, we conclude that *NETO1* should be added to the list of risky genes that should be investigated for ASD.

## Conclusion

Our data expand the spectrum of 22q11.2 region mutations and provide insights for genotype–phenotype correlations of ID, GDD, and autism. It also underlines the importance of CMA and its use in understanding the pathophysiology of certain diseases. Based on our findings, we suggest that CMA should be used as a first-step test for the identification of new loci and the expansion of known phenotypes.

## Abbreviations

ASD: Autism spectrum disorder
CA: Congenital anomalies
CMA: Chromosomal microarray analysis
CNVs: Copy number variations
DQ: Dosage quotient
GDD: Global developmental
ID: Intellectual disability
MLPA: Multiplex ligation-dependent probe amplification
UCS: Uncertain clinical significance
